# Skeletal Muscle Myofibrillar and Sarcoplasmic Protein Synthesis Rates Are Affected Differently by Altitude-Induced Hypoxia in Native Lowlanders

**DOI:** 10.1371/journal.pone.0015606

**Published:** 2010-12-20

**Authors:** Lars Holm, Mads Lyhne Haslund, Paul Robach, Gerrit van Hall, Jose A. L. Calbet, Bengt Saltin, Carsten Lundby

**Affiliations:** 1 Department of Orthopaedic Surgery M and Institute of Sports Medicine, Bispebjerg Hospital, and Faculty of Health Sciences, Center of Healthy Ageing, University of Copenhagen, Copenhagen, Denmark; 2 Copenhagen Muscle Research Center, Rigshospitalet, Copenhagen, Denmark; 3 Ecole Nationale de Ski et D'Alpinisme, Chamonix, France; 4 Metabolic Mass-Spectrometry Facility, Rigshospitalet, and Department of Biomedical Sciences, Faculty of Health Sciences, University of Copenhagen, Copenhagen, Denmark; 5 Department of Physical Education, University of Las Palmas de Gran Canaria, Las Palmas de Gran Canaria, Canary Islands, Spain; Flinders University, Australia

## Abstract

As a consequence to hypobaric hypoxic exposure skeletal muscle atrophy is often reported. The underlying mechanism has been suggested to involve a decrease in protein synthesis in order to conserve O_2_. With the aim to challenge this hypothesis, we applied a primed, constant infusion of 1-^13^C-leucine in nine healthy male subjects at sea level and subsequently at high-altitude (4559 m) after 7–9 days of acclimatization. Physical activity levels and food and energy intake were controlled prior to the two experimental conditions with the aim to standardize these confounding factors. Blood samples and expired breath samples were collected hourly during the 4 hour trial and vastus lateralis muscle biopsies obtained at 1 and 4 hours after tracer priming in the overnight fasted state. Myofibrillar protein synthesis rate was doubled; 0.041±0.018 at sea-level to 0.080±0.018%⋅hr^−1^ (p<0.05) when acclimatized to high altitude. The sarcoplasmic protein synthesis rate was in contrast unaffected by altitude exposure; 0.052±0.019 at sea-level to 0.059±0.010%⋅hr^−1^ (p>0.05). Trends to increments in whole body protein kinetics were seen: Degradation rate elevated from 2.51±0.21 at sea level to 2.73±0.13 µmol⋅kg^−1^⋅min^−1^ (p = 0.05) at high altitude and synthesis rate similar; 2.24±0.20 at sea level and 2.43±0.13 µmol⋅kg^−1^⋅min^−1^ (p>0.05) at altitude. We conclude that whole body amino acid flux is increased due to an elevated protein turnover rate. Resting skeletal muscle myocontractile protein synthesis rate was concomitantly elevated by high-altitude induced hypoxia, whereas the sarcoplasmic protein synthesis rate was unaffected by hypoxia. These changed responses may lead to divergent adaptation over the course of prolonged exposure.

## Introduction

Long-term (75 days) exposure of healthy lowlanders to high-altitude (5250 m at Mount Everest base camp) induces loss of skeletal muscle mass and single fiber atrophy in m. vastus lateralis and m. biceps brachii [1]. Similar results have been obtained in subjects residing for 40 days in a hypobaric chamber [2]. In mountaineers climbing in the Himalayas severe loss >20% of single fiber area has been reported in biopsies obtained upon return to sea level [3], [4]. Thus, hypoxic exposure has in several studies been shown to be detrimental for skeletal muscle mass. However, contrasting results have also been reported. For example, following 56 days at 4100 m altitude no significant changes in single fiber cross sectional area was observed [5]. Besides the lower residing altitude in this study, subjects had free access to physical activities such as soccer, hiking, and jogging, which may have counteracted the severity of muscle loss and thus its detection. Following a 21-day expedition culminating with the assent to 6194 m, six mountaineers experienced no significant muscle loss [6]. Thus, total time of exposure, the degree of hypoxia, physical activity levels, and food intake are all factors, which may markedly influence the adaptation of skeletal muscle to hypoxia.

In studies, applying indirect measures of protein turnover, *in vitro* models provide results that suggests that attenuated energy turnover and loss of muscle mass may be developed at hypoxia exposure due to a decrease in overall protein turnover rate [7]–[10]. Surprisingly, Vigano and co-workers observed no marked changes in translation regulating following 7–9 days of stay at 4559 m altitude [11]. Exemplified by the total mammalian target of rapamycin (mTOR), a master regulator of protein synthesis, they found a slight decrease in its protein content, unfortunately with no measures of its phosphorylation status or activity [11]. The protein expression and phosphorylation state of the eukaryotic translation initiation factor 2 alpha (eIF2α) was unchanged [11], which was also the case for the protein concentration of the hypoxia inducible factor (HIF). In a previous publication however, the HIF gene was shown to be markedly increased at the transcriptional level [12].

A direct measurement of the protein kinetics processes is though more valid to decide whether altitude affects protein turnover. Using the stable isotope methodology, Imoberdorf and co-workers assessed muscle protein synthesis rate after exposure to high altitude [13]. They showed that in a group of subjects, investigated acutely after active ascent to high-altitude, mixed muscle protein synthesis rate was higher compared to in a group that was flown to the altitude and compared to the rate at sea level [13]. In this study the measurements may have been affected by the differences in ascent modality and the short acclimatization time allowed at altitude prior to measurements. Hence, from cell culture experiments it is known that some metabolic changes to hypoxic exposure takes place in a time-dependent manner; such as decrease in cellular ATP concentration and a decrease in oxygen consumption to protein synthetic processes [7], which means that some exposure time may be needed to allow the adaptation to occur.

We therefore hypothesized that skeletal muscle protein synthesis could be affected by high-altitude induced hypoxia if longer exposure time would have been allowed. Therefore, to study whole body and skeletal muscle protein kinetics, we completed trials with stable isotopically-labeled amino acid infusions at sea level and 7–9 days after ascent to high altitude (4559 m).

## Methods

### Subjects

Nine healthy, physically active male subjects participated in the study. They ranged in age from 24 to 30 years, had an average height of 180±1 cm (mean ± SEM), and a mean weight of 78±2 kg. Not all data points were obtained in all subjects during the altitude trials, and the exact n is given in tables/figures where needed. Results describing altitude induced changes in skeletal muscle proteins involved in iron transport, tricarboxylic acid (TCA) cycle, oxidative phosphorylation, and oxidative stress responses from the same study have been published elsewhere [11], [12].

### Ethics Statement

The study conformed with guidelines laid down in the Declaration of Helsinki II and was approved by the Copenhagen and Frederiksberg Ethics Committee, Denmark. After being given both written and oral information on the experimental protocol and procedures, the subjects gave their informed, written consent to participate.

### Experimental design

In April all subjects reported to the laboratory at sea level in Copenhagen three days prior to the experiments and were not allowed to leave the building until experiments had finished. Most of the time was passed watching TV, studying, working on computers, or conversation. During their indoor confinement the subjects had access to a broad variety of food and the subjects choose themselves what to eat at all times. All food intakes were registered. The day before the experiment no caffeine containing substances was allowed from 6 pm, and the subjects fasted from 8 pm until after completion of the experiments the following day. The next morning at 8 am the experiments were initiated and the protocol is described in the paragraph *Experimental protocol*.

In late August all subjects were flown to Italy and brought to 3200 m altitude by bus and lift. After 2–3 hours of mountain walking all subjects arrived at the Gnifetti hut at 3647 m and spend two nights in order to minimize the risk of acute mountain sickness (assessed by Lake Louise scores). The experimental facilities of the Margeritha hut (4559 m altitude) were reached after 5–7 hours of mountain/glacier walking on day 3 and subjects resided here until the experiments were started 7–9 days after arrival to this altitude. The Margeritha hut is a modern building where parts of the second floor are allocated specifically for scientific projects. The building is well insulated, has electricity, and comfortable living and kitchen facilities, and the experimental settings cannot be compared to the previous studies conducted in for example the base camp of Mount Everest where low temperatures etc may influence the results.

As at sea level, all subjects were confined to indoor activities three days prior to the experiments and were not allowed outside in an attempt to standardize energy expenditure. We had brought an exactly matched amount of food as the subjects ate during the sea level experiments and the subjects consumed a similar diet as they did at sea level during this period. The restrictions to food prior to the experiments were the same as they were initiated at sea level.

### Arterial blood sample

Approximately 10 days after the completion of the experiments at sea level an arterial blood sample was taken for other purposes [14]. Two days after the subjects had completed the experimental trials at altitude, an arterial blood sample was obtained while still at altitude. *Experimental protocol*

Subjects were studied overnight fasting and in the supine position. A 20 G venflon (BD Venflon, Sweden) was inserted in an antecubital vein in both arms, and one was used for infusions and the other for blood sampling. After a priming dose of 1-^13^C-leucine (7.0 µmol·kg^−1^) added H^13^CO_3_ (2.5 µmol·kg^−1^) to label the carbondioxide pool with ^13^C as well, the constant infusion of 1-^13^C-leucine (0.15 µmol·kg^−1^·min^−1^) was initiated and continued for 4 hours. The stable isotope tracers were sterility and pyrogenity tested and 99% enriched and purchased from Cambridge Isotopes Laboratories (Andover, MA, USA). They were dissolved in sterile saline prior infusion and sterilized through a 0.2 µm disposable filter (Sartorius, Hannover, Germany). Muscle biopsies were obtained prior to any infusions and after 1 and 4 hours of infusions. Biopsies were obtained from vastus lateralis muscle through a new incision after local anesthesia (1% lidocaine) using a 5 mm Bergstrom needle with suction and were always obtained from the same, non-dominant leg.

Before and one day after return to sea level all subjects were scanned for body composition in a dual-energy X-ray absorptiometer (DEXA; Lunar, GE Medical Systems, Madison, WI, USA). For more detailed subject characteristic see reference [14].

### Measurements

Blood samples were obtained pre infusion and at every hour during the infusion in EDTA containing tubes and was centrifuged to obtain plasma, which was stored at –80°C for later analysis of hormone concentrations and stable isotope abundances. Whole body O_2_ uptake (VO_2_), CO_2_ production (VCO_2_), and expired minute ventilation (V_E_) were measured for 15 minutes pre infusion and at every hour during the infusion (Quark b^2^, Cosmed Srl., Rome, Italy). Based on ambient conditions on the experimental days the gas analyser and the flowmeter were calibrated with high precision gases. Expired air was also collected in a Douglas bag and transferred to vacuum glass containers (BD Vacutainer Systems, Plymouth, UK) for later analysis of ^13^CO_2_ enrichments.

### Analysis

Plasma concentrations for insulin (kit: DYC1544-2), human growth hormone (kit: DGH00), cortisol (kit: KGE008), and testosterone (kit: KGE010) were assessed by available ELISA kits (R6D Systems, Minneapolis, MN, USA) and insulin-like growth factor-1 (ELH-IGFBP1-001) with ELISA kits (RayBio, Norcross, GA, USA) applying the prescribed procedures.

The ^13^C-leucine tracer concentration in the infusate was determined using the internal tracer standard method: adding a known amount of L-[5,5,5-D_3_]-leucine to the sample and relating the fragment peak of [M+3] to the infused isotope peak [M+1] with M equal to m/z = 302 based on the obtained GC/MS chromatograms (gas chromatograph; Trace GC 2000 series and mass spectrometer; Automass Multi, Thermo Quest Finnigan, Paris, France) using a capillary column (CP-Sil 8 CB low bleed 30 m×0.32 mm, coating 0.25 µm, ChromPack, Varian, Palo Alto, CA) to isolate leucine and the EI-mode to create the fragment-ions. Based on the pump recorded infusion speed and the measured leucine tracer concentration, the delivery of leucine tracer to the subjects was calculated.

Breath CO_2_ was analyzed for ^13^C abundance originating from oxidation of the ^13^C-leucine tracer on the IRMS system (isotope ratio mass spectrometry, Delta Plus XL, Thermo Finnigan, Bremen, Germany) with CO_2_ separated by a CP-PoraPLOT Q column, 27.5 m×0.32 mm×10 µm (ChromPack, Varian, Palo Alto, CA).

The surrogate measure of the precursor pool for protein synthesis with leucine as the traced amino acid, plasma ^13^C-ketoisocaproic acid (KIC) abundance was used [15] and measured at GC/MS system (gas chromatograph; Trace GC 2000 series and mass spectrometer; Automass Multi, Thermo Quest Finnigan, Paris, France). A capillary column (CP-Sil 8 CB low bleed 30 m×0.32 mm, coating 0.25 µm, ChromPack, Varian, Palo Alto, CA) was used to separate the compound and that was analyzed using the EI-mode to fragment the molecule measuring on the ion m/z = 232. Briefly, plasma proteins were precipitated from 140 µL plasma with ethanol and the supernatant was dried under N_2_ at 50°C. With 200 µL acidified 2% w/v o-phenylenediamine in 200 µL Millipore water the substrates were prepared for the derivatization using pyridine and BSTFA +1% TMCS (#38831, Pierce, Bie & Berntsen & VWR International, Rødovre, Denmark).

The protein-bound leucine abundance was measured by GC-C-IRMS system (chromatography–combustion–isotope ratio mass spectrometry, Delta Plus XL, Thermo Finnigan, Bremen, Germany), where the leucine was isolated by a capillary column (CP-Sil 19 CB 60 m×0.25 mm, coating 1.5 µm, ChromPack, Varian, Palo Alto, CA). The myofibrillar and sarcoplasmic protein-fractions were separated from a muscle homogenate using a common solubility/centrifugation procedure [16]. After homogenized in buffer (0.15 M NaCl, 5 mM EDTA, 0.1% Triton X-100, and 0.02 M Tris (pH 7.4)) using an automatic FastPrep homogenizer (model 120A-230, Thermo Savant, Holbrook, NY), the myofibrillar and collagenous proteins were precipitated by centrifugation (1600 g, 4°C, 20 min). From the supernatant, the sarcoplasmic proteins were precipitated with 2.3× vol ice-cold ethanol and spun (1600 g, 4°C, 20 min). The myofibrillar proteins were resuspended in 0.7 M KCl and after centrifugation (1600 g, 4°C, 20 min) they were subsequently precipitated from the supernatant with 2.3× vol ice-cold ethanol. Protein pellets were then hydrolyzed in 6 M HCl (110°C for 18 h). The constituent amino acids were purified by cation exchange resin (Dowex AG-50W, Bio-Rad, Copenhagen, Denmark) and derivatized as their *N*-acetyl *n*-propyl (NAP) esters.

### Calculations

All whole body ventilation and leucine-based results are reported as the mean of data obtained at 3 and 4 hours whereas leucine incorporation into myofibrillar and sarcoplasmic protein fractions is determined from 1–4 hours of infusion. Intracellular leucine is in equilibrium with its transaminated product, the alpha-ketoisocaproic acid (KIC). Since the leucine metabolism occurs at the intracellular site, the venous enrichment of ^13^C-KIC more accurately reflects the intracellular abundance of ^13^C-leucine than the venous ^13^C-leucine [15].

Whole body leucine rate of appearance (R_a, leu_), which is an estimate of whole body protein breakdown, was calculated using: Infusion rate_tracer_ ⋅ E_KIC, estimate of intracellular tracer_^−1^.

The underlying assumption for the applied tracer calculations is that steady state is present for the tracer kinetics, i.e. leucine rate of appearance, a measure of whole body leucine release R_a, leu_ = R_d, leu_ (leucine rate of disappearance, an estimate of whole body disposal of leucine). Since leucine can be oxidized, not all of the ‘disappearing’ leucine goes for synthesis. Therefore, non-oxidative whole body leucine rate of disappearance (NOLD) rate, which is an estimate of whole body protein synthesis, is calculated: NOLD_leu_ = R_d, leu_ – Ox_leu_. Leucine oxidation rate (Ox_leu_) was calculated using the following equation: E_CO2_ ⋅ CO_2_ production rate ⋅ bicarbonate correction factor 0.81 ⋅ E_KIC, estimate of intracellular tracer_^−1^, where the bicarbonate correction factor of 0.81 is a standard value for the detainment of CO_2_ in the metabolism [17]. The CO_2_ production rate was calculated using the equation: VCO_2_ ⋅ (22.4 ⋅ Body weight)^−1^ ⋅ 1000, where the CO_2_ production was found by analyzing the expired gas collected in the Douglas bags (nondiffusible Douglas bag, Hans Rudoph).

The protein fractional synthesis rate (FSR) was calculated using the precursor-product equation: FSR = ΔE_product_⋅E_precursor_^−1^⋅Δtime^−1^, where ΔE_product_ is the change in ^13^C-leucine tracer enrichment in two tissue samples taken with a time interval of Δtime hours. E_precursor_ is the average precursor pool enrichment during Δtime hours determined by the valid surrogate measure of venous ^13^C-KIC enrichment [15].

The group-mean ^13^C-KIC-enrichments, used as a valid estimate of the protein synthesis precursor pool ^13^C-leucine-enrichment [15], [18], were found to be slightly higher towards the end of the tracer-incorporation period than in the beginning. Such minor fluctuations and changes in tracer steady state are often seen in tracer studies applying the primed-continuous infusion protocol, as the perfect priming is hard to calculate. When using ^13^C-leucine as a tracer and venous ^13^C-KIC as a derived estimate of the intracellular tracer abundance, those are thought to be equilibrated during such minor drifts in tracer abundance and thus are ascribed to reflect the actual precursor enrichment. Thus, by using a weighted mean of the precursor pool enrichment the minor drift towards higher enrichments is not supposed to violate the underlying assumptions for the FSR calculations.

### Statistics

Values obtained at sea level and at high altitude were compared by a parametric Students paired t-tests. The precursor enrichments at the single time points were compared by a two-way ANOVA with repeated measures. One subject could not complete the infusion trial at high altitude, thus total number of skeletal muscle protein synthesis rates is eight. Due to technical problems with the gas collections the whole body protein kinetics calculated from the leucine tracer data only exist for six subjects. Values are reported as mean ± SEM. Statistical significance was obtained when the p-value was <0.05. Analyses were performed in Prism 4.0 (GraphPad software, San Diego, CA, USA).

## Results

### Subject characteristics

High altitude exposure did not influence body weight (78.3±2.3 to 77.9±2.9 kg), resting metabolic rate (212±32 to 221±22 mL·min^−1^) or VO_2_max measured before high altitude exposure and one day after return to sea level (4.4±0.1 and 4.4±0.1 L·min^−1^, or 56.2±2.0 and 56.5±2.1 mL·kg^−1^·min^−1^, respectively). DEXA scans revealed that also the body composition was not altered, i.e. at sea level and high-altitude fat mass was 13.4±4.2 and 13.2±4.5 kg, respectively and lean body mass was 63.7±6.1 and 63.6±6.1 kg, respectively.

### Arterial blood gasses

Hemoglobin concentration was increased from 144±8 to 169±13 g·L^−1^ from sea level to altitude, and PaCO_2_ reduced from 40.4±2.0 to 29.2±1.9 mmHg. Likewise PaO_2_ and SaO_2_ were decreased from 103.2±13.8 and 98.6±0.5 to 50.5±3.2 mmHg and 83.4±2.7%, respectively. As a consequence of these changes, arterial O_2_ content remained unchanged and was 199±10 at sea level and 191±18 mL·L^−1^ at altitude.

### Whole body protein turnover determined with the leucine tracer

Resting and fasting whole body leucine rate of appearance (R_a_), an estimate of protein degradation, tended to be increased at high altitude compared to sea level (Table 1, p = 0.05, n = 6). Further, non-oxidative leucine disappearance (NOLD), an estimate of protein synthesis, and leucine oxidation also showed a numerical increase at altitude although the change did not reach statistical significance (Table 1, p = 0.19 and p = 0.16, respectively, n = 6).

**Table 1:** Whole body leucine turnover.

	Sea level	Altitude	p value
Whole body leucine Ra	2.51±0.21	2.73±0.13	0.05
Leucine oxidation	0.27±0.02	0.31±0.01	0.16
Non-oxidative leucine Rd	2.24±0.20	2.43±0.13	0.19

Whole body leucine rate of appearance (Ra), leucine oxidation, and non-oxidative leucine rate of disappearance (Rd) [µmol⋅kg^−1^⋅min^−1^] in male subjects at sea level and after 7–9 days exposure to high altitude. N = 6, and values are mean ± SEM.

### Muscle protein synthesis rate

**Figure 1:**
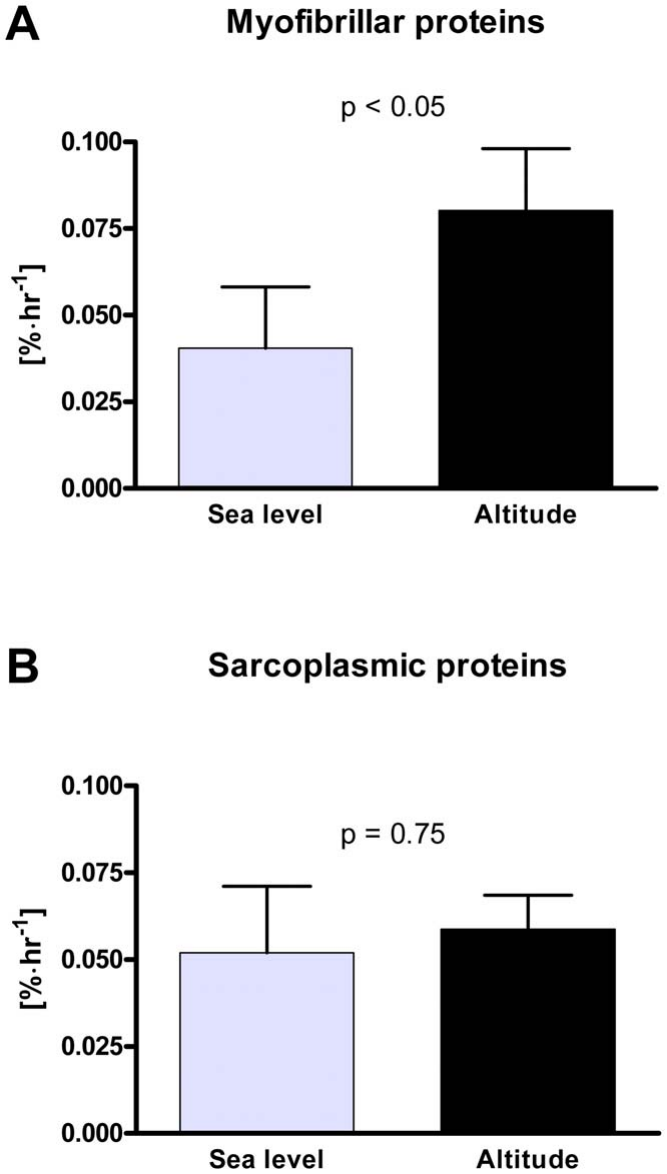
Muscle protein fractional synthesis rate. Resting and fasting fractional synthesis rate [%·hr^−1^] of A) myofibrillar proteins and B) sarcoplasmic proteins at sea level and after 7–9 days of acclimatization to 4559 m altitude. N = 8 and values are mean ± SEM.

Enrichment of the surrogate measure of muscle protein synthesis precursor estimated by the venous plasma ^13^C-KIC enrichments is reported in Table 2. A slight increase over the course of the FSR-determination from 1 to 4 hours after start of infusion (p<0.001) was seen as well as an effect of altitude (p<0.05) with the enrichment at altitude being 13% lower than at sea level. Vastus lateralis muscle myofibrillar protein fractional synthesis rate in the resting, overnight fasted state was enhanced from 0.041±0.018 at sea level to 0.080±0.018%·hr^−1^ following 7–9 days of high altitude acclimatization (p<0.05, Figure 1A), whereas the sarcoplasmic protein synthesis rate was unchanged by altitude exposure, 0.052±0.019%·hr^−1^ at sea level and 0.059±0.010%·hr^−1^ at altitude (p>0.05, Figure 1B).

**Table 2:** Venous plasma KIC and muscle protein bound leucine enrichments.

		1 hr	2 hr	3 hr	4 hr
Sea level	Venous plasma ^13^C-KIC	5.70±0.16	6.13±0.20	6.33±0.22	6.57±0.25
	Myofibrillar protein	1.0835±0.0003			1.0844±0.0005
	Sarcoplasmic protein	1.0886±0.0004			1.0898±0.0005
Altitude	Venous plasma ^13^C-KIC	5.05±0.26	5.27±0.21	5.58±0.19	5.74±0.22
	Myofibrillar protein	1.0845±0.0005			1.0861±0.0007
	Sarcoplasmic protein	1.0894±0.0001			1.0905±0.0002

Enrichments in the precursor; venous plasma alpha-^13^C-ketoisocaproic acid (KIC) in percent tracer-to-tracee ratio (TTR) at time points every hour throughout the FSR-determination period. An effect of time (p<0.001) and altitude (p<0.05) appeared. Isotope ratios of ^13^C/^12^C in leucine liberated from the myofibrillar and sarcoplasmic protein fractions, respectively, in the muscle tissue at 1 and 4 hours at sea level and at altitude. N = 8 and values are mean ± SEM.

### Hormones

Plasma concentrations of human growth hormone, cortisol, testosterone, and insulin-like growth factor I (IGF-I) remained unchanged with altitude exposure, whereas insulin concentrations increased from 4.9±0.4 to 7.7±1.1 µlU·mL^−1^ (p<0.05, Table 3).

**Table 3:** Venous plasma hormonal concentrations.

	Sea level	Altitude	p value
Insulin	4.7±0.4	7.7±1.1	<0.05
hGH	2.7±0.7	1.5±0.1	Ns
IGF-I	141±9	151±14	Ns
Testosterone	13.1±2.2	17.4±2.9	Ns
Cortisol	88.3±15.4	89.3±13.0	Ns

Venous plasma concentrations of insulin [µlU·mL^−1^], human growth hormone [hGH; µlU·mL^−1^], cortisol [ng·mL^−1^], testosterone [pg·mL^−1^], and insulin like growth factor-1 [ng·mL^−1^] in male subjects at sea level and after 7–9 days exposure to high altitude. N = 9 and values are mean ± SEM.

## Discussion

Loss of skeletal muscle is a common observation when healthy native lowlanders are exposed to high altitude for prolonged periods [19]. Furthermore, the mean fiber area in the vastus lateralis muscle of Aymara Bolivian natives residing in a La Paz at 4100 m is significantly smaller than in native European lowlanders [5]. Although the atrophic phenomenon at altitude has been described for decades, the underlying peripheral protein kinetics and mechanisms remain unresolved and only few human data on protein turnover in response to hypoxia exist. The main finding in the present study was that resting, fasting skeletal muscle sarcoplasmic protein synthesis rate is unchanged after 7–9 days acclimatization to altitude whereas the contractile protein synthesis rate is markedly elevated (Figure 1).

The synthesis rate of human skeletal muscle protein has only sparsely been assessed during hypoxic conditions and data are somehow contrasting. Imoberdorf and co-workers assessed the fractional synthesis rate of mixed skeletal muscle protein at sea-level and divided the subjects into two groups subsequently; one group that was flown to high altitude and another that walked from 3220 m up to 3611 m (1.5 hr) on one day and then to 4559 m (4–5 hrs) the next day [13]. Acutely (19–24 hrs) after arrival at the high altitude destination the trials were conducted, which revealed an unchanged resting synthesis rate of the mixed muscle proteins in the passively ascended group and an elevated synthesis rate in the walking group [13]. Although well-conducted, the study has two critique points in terms of evaluating how hypoxia affects skeletal muscle protein synthesis rate. First, performing the trials shortly after arrival presumably does not allow sufficient time for relevant hypoxic responses to occur and thus does not determine whether chronic hypoxia affects protein synthesis rate. In this regard, rodent studies reveal that the adaptive response in muscle protein turnover to hypoxic conditions is affected by time [20], [21]. Secondly, the elevated muscle synthesis rate in the active group may simply be caused by the physical activity (active ascent) [22], [23] conducted one and two days prior the experimental trial [13], which is a bias with regards to the hypoxic exposure intervention. In the other human study examining the response of hypoxia on protein metabolism, the forearm muscle compartment was shown to slower its protein synthesis (estimated by leucine uptake) with acute exposure to hypobaric hypoxia [24]. These divergent results also signify the importance of evaluating protein kinetics at more protein specific level, since different protein fractions may diverge in responsiveness. Therefore, not necessarily in contrast to previous results [13], [24], the myocontractile protein synthesis rate was elevated after ∼1 week of acclimatization to high altitude (Figure 1A). As the problem during hypoxic exposures generally is loss of skeletal muscle mass, the enhanced synthesis rate of the contractile proteins must be accompanied by an even higher increase in the protein breakdown rate. That a similar stimulus may have been applied to the present subjects is implied by the trend toward an increase in the whole body protein degradation rate (Ra, Table 1). In the same subjects however, Vigano et al. reported that the concentration of the muscle myofibrillar protein actin was unchanged with altitude exposure [11]. Therefore, the measured overall elevation in protein turnover with hypoxia exposure, which also is likely to have been the case for the myofibrillar protein breakdown rate, may have been counterbalanced by a concomitant increase in the myofibrillar protein synthesis rate. Thereby, resulting in a steady actin protein concentration and an undetectable change in muscle mass [11].

In contrast, we found an unaffected synthesis rate of the sarcoplasmic proteins in skeletal muscle (Figure 1B). Using a similar muscle protein separation protocol, it has been verified that the sarcoplasmic protein fraction contains water soluble proteins present intracellularly as well as subsarcolemmal mitochondrial proteins [16], [25]. Assuming that the overall protein turnover rate is enhanced, an absence of enhanced protein synthesis rate will result in net protein breakdown leading to net protein loss. For the sarcoplasmic proteins this may be the situation and this is in agreement with the finding from the same muscle specimens showing a decreased concentration of various metabolic enzymes and stress responsive proteins [11] as well as several proteins involved in the oxygen transport [12], of which most presumably will be present in the sarcoplasmic protein fraction. Although, the decrements in protein concentrations in these studies were unexpected for the authors at that time, because most of the mRNAs coding for those proteins were more concentrated at altitude [12], the findings are in agreements with decreased mitochondrial density and content of metabolic enzymes after prolonged exposure to hypoxia shown in other studies [4], [26], [27]. Relating to these long-term adaptations, it can therefore be suggested that the breakdown rate of the proteins in the sarcoplasmic protein fraction as well as for other proteins in the body is enhanced, which thereby in the absence of elevated synthesis rate results in a negative net sarcoplasmic protein balance, leading to protein loss. We did not observe any indications of altered oxidative capacity with hypoxic exposure in the present study (similar submax and max VO_2_ at sea level and with acute normoxia at altitude). Whether, the turnover rates of the specific proteins leading to the metabolic changes and decreased oxidative capacity were affected by hypoxia or not or the changes yet were unable to exert functional changes are unknown. As tempted to outline, several adaptations have to take place along the path from changes in gene and protein expression till detectable physiological manifestations.

Venous plasma KIC is not solely originating from skeletal muscle, but is released from all cells making the venous plasma KIC a weighted mean of whole body intracellular leucine enrichments. It is generally agreed that since skeletal muscle make up such an abundant tissue, the venous plasma KIC enrichments is highly reflective of the status in the myocytes [15], [18]. Interestingly, we observed a 13% lower enrichment in the KIC at altitude compared to at sea level (Table 2). Since the enrichment at steady state is defined as the tracer infusion rate divided by the rate of appearance of the tracee, the lower enrichment at altitude is presumably due to a higher rate of appearance and thus flux of free leucine at altitude. This observation thus coincides with the trend toward increased whole body leucine flux and significant increase in leucine rate of appearance (Table 1), which is a measure of protein degradation. Unfortunately, we do not have any tissue or protein specific data for degradation, which would have gained valuable information in combination with the synthesis results. However, protein specific methods to assess the protein breakdown rate comparable to the fractional synthesis rate approach await yet to be developed.

It is not believed that the observed protein turnover is driven by changes in systemic hormones as we (Table 2) and others [13], [28] have not been able to show changes in a range of potent hormones except from insulin, which previously has been found to be increased after hypoxic exposure [28]. Both circulating glucose and insulin is elevated after acclimatization to high altitude [28], [29], indicating a diminished insulin sensitivity under hypoxic conditions. It is unlikely that this relatively small increase in insulin should be responsible for the observed changes in skeletal muscle protein turnover [30].

It has previously been demonstrated that the hypoxic response may not be as detrimental for the cell [5] as the *in vitro* conditions, which lead to the formulation of the “Hypoxia Defence Strategies” by Hochachka and co-workers [8]. Thus, a less severe response than the initiation of the two-phase *Defence and Rescue* strategy may be expected. During an exposure to a mild hypoxic milieu as in the present setting, we propose a remodeling adaptive response in the skeletal muscle, which initiates an increase in protein degradation. Subsequently, it is left to the synthetic processes to reorganize and rebuild the tissue. Whether the net outcome over time then ends up with a steady or slightly decreased protein mass is depended on how well the protein synthetic processes are maintained. The stimulation of protein synthesis may primarily be through external factors, which at altitude may be compromised by decreasing food intake, decrease in physical activity level [19], gastric disorder, minor inflammatory states [31], [32], and various other high-altitude related inconveniences [33]. The net muscle protein balance may thereby be susceptible to become negative leading to a net loss of muscle mass as it is observed in most but not all high-altitude studies. Further, when the protein turnover rate is high, the protein mass is more vulnerable to change when small imbalances in net balance appear. It is known though, that skeletal muscles maintain their capacity to adapt to endurance training at altitude [34]. Therefore, if the compromising factors for the stimulation of protein synthetic processes are not sought treated and counteracted at high altitude a negative net protein balance seem unavoidable. In the present study, we sought to match energy expenditure at altitude with those at sea level and provided the subjects with excess food, which in our setting may have maintained the myofibrillar protein synthesis high. Assuming an overall increased release of amino acids after several days of high altitude acclimatization, the lack of elevated synthesis rate of the sarcoplasmic and mitochondrial proteins therefore coincides with the ubiquitously observed decrease in oxidative capacity following exposure to high altitude.

The applied tracer approach validly reveals that the adaptation of synthesis rates of various skeletal muscle protein fractions is different and future hypoxia-research should focus on describing the molecular signaling responsible for these divergent adaptations. With regard to the measurement of protein breakdown rate, no similar protein specific approach exists. However, the whole body amino acid flux rates assessed here indicate an elevation at altitude when compared to sea level but these results are underpowered and not protein specific. Therefore, the adaptation and regulation of the protein breakdown after acclimatization to high altitude demands thorough research and improvements in methodology.
